# Performance and cross-cultural comparison of the short-form version of the CPQ_11-14 _in New Zealand, Brunei and Brazil

**DOI:** 10.1186/1477-7525-9-40

**Published:** 2011-06-07

**Authors:** Lyndie A Foster Page, W Murray Thomson, A Rizan Mohamed, Jefferson Traebert

**Affiliations:** 1 Department of Oral Rehabilitation, School of Dentistry, University of Otago, New Zealand; 2 Department of Oral Sciences, School of Dentistry, University of Otago, New Zealand; 3 Department of Oral Health, Ministry of Health, Brunei Darussalam; 4 Post-Graduate Programme in Health Sciences, Southern Santa Catarina University, Florianópolis, Brazil

**Keywords:** Adolescents, caries experience, quality of life, validity, short-form CPQ_11-14_

## Abstract

**Background:**

The Child Perception Questionnaire (CPQ_11-14_) is a self-report instrument developed to measure oral-health-related quality of life (OHRQoL) in 11-14-year-olds. Earlier reports confirm that the 16-item short-form version performs adequately, but there is a need to determine the measure's validity and properties in larger and more diverse samples and settings.

**Aim:**

The objective of this study was to examine the performance of the 16-item short-form impact version of the CPQ_11-14 _in different communities and cultures with diverse caries experience.

**Method:**

Cross-sectional epidemiological surveys of child oral health were conducted in two regions of New Zealand, one region in Brunei, and one in Brazil. Children were examined for dental caries (following WHO guidelines), and OHRQoL was measured using the 16-item short-form item-impact version of the CPQ_11-14_, along with two global questions on OHRQoL. Children in the 20% with the greatest caries experience (DMF score) were categorised as the highest caries quintile. Construct validity was evaluated by comparing the mean scale scores across the categories of caries experience; correlational construct validity was assessed by comparing mean scores and children's global ratings of oral health and well-being.

**Results:**

There were substantial variations in caries experience among the different communities (from 1.8 in Otago to 4.9 in Northland) and in mean CPQ_11-14 _scores (from 11.5 in Northland to 16.8 in Brunei). In all samples, those in the most severe caries experience quintile had higher mean CPQ_11-14 _scores than those who were caries-free (P < 0.05). There were also greater CPQ scores in those with worse self-rated oral health, with the Otago sample presenting the most marked gradient across the response categories for self-rated oral health, from 'Excellent' to 'Fair/Poor' (9.6 to 19.7 respectively).

**Conclusion:**

The findings suggest that the 16-item short-form item impact version of the CPQ_11-14 _performs well across diverse cultures and levels of caries experience. Reasons for the differences in mean CPQ scores among the communities are unclear and may reflect subtle socio-cultural differences in subjective oral health among these populations, but elucidating these requires further exploration of the face and content validity of the measure in different populations.

## Introduction

The CPQ_11-14 _is a self-report questionnaire developed to measure oral health-related quality of life in children and adolescents [1]. The original CPQ_11-14 _comprised 37 items organised into four health domains. It is usually administered with two additional items related to the child's global rating of his/her oral health; these serve as a validity check. Items for the CPQ_11-14 _were selected using an item impact study which identified items of most importance to the patient population [1]. It was validated by using a clinical convenience sample [1] and a population sample [2], and has been cross-culturally adapted for use in a number of cultures and languages [3-7]. The questionnaire's length and the associated respondent burden were thought to limit its routine use in dental epidemiology and health services research. The development of a short-form CPQ_11-14 _was thought to broaden its application, by decreasing the likelihood of unit or item non-response and reducing respondent burden. Jokovic and co-workers developed four short-form versions of the CPQ_11-14 _using two different approaches [8-10]. This resulted in two 8-item versions and two 16-item versions.

The initial work using a clinical convenience sample showed that all four short-forms detected substantial variability in children's OHRQoL, with the 16-item questionnaires being almost identical, and the 8-item questionnaires only just differing [11]. Further validation of the short-form versions was provided in a study of a random population sample of New Zealand adolescents who had completed the full questionnaire. This work confirmed that all short-form versions showed acceptable properties, but that the 16-item versions performed better [12]. These findings were then confirmed in a Brazilian convenience sample of 11- to 14-year-olds, who had been assigned to three groups (healthy, caries present, malocclusion present) after being examined. This study, being the first to administer only the short-form questionnaire (rather than the longer version), provided evidence of the satisfactory properties (reliability and construct and discriminant validity) of the Brazilian version, although the 16-item version performed better than the 8-item one [13]. While the various studies findings on the short-form version support its validity, there has been substantial variation in mean CPQ scores [11,12]. These OHRQoL differences and the reason for them have not been reported on. There is a need to determine the shortened measure's validity and properties in larger and more diverse samples and settings before any recommendations on its future use can be made.

The objective of this study was to further examine the performance of the 16-item short-form impact version of the CPQ_11-14 _in different communities and cultures with diverse caries experience, and to compare the subjective oral health of these different communities.

## Method

Data from studies of children in New Zealand (Northland and Otago), Brunei and Brazil were used in this study. Each is briefly described below. All studies used the short-form 16-item impact version of the CPQ_11-14 _[12,13]. Two global questions on OHRQoL were also reported. First, participants were asked to rate the health of their teeth, lips, jaws and mouth; and second, they were asked how much their teeth, lips, jaw or mouth affects their life overall. Sociodemographic information was collected. All studies carried out dental caries examinations (following World Health Organization guidelines) using calibrated public-sector dentists [14].

### Northland

A cross-sectional epidemiological survey was conducted of all 12- and 13-year-old children attending schools in 2008. Ethnicity was obtained from the children's parents and was classed as Māori or non-Māori. We also recorded the school "decile rating", the New Zealand Ministry of Education's targeted funding for educational achievement (TFEA) indicator for schools [15], which is an area-based socio-economic status (SES) measure which allocates scores ranging from 1 (lowest SES) to 10 (highest SES) to schools. For intra-examiner reliability, the intraclass correlation coefficient for DMFS was 1.00; for inter-examiner reliability, it was 0.98. Ethical approval for the study was obtained from the Northern Y Regional Ethics Committee.

### Otago

A cross-sectional epidemiological survey was conducted of all 12- and 13-year-old children attending intermediate schools in Dunedin in 2010. Ethnicity and socio-economic data were obtained from the parent. Ethnicity was classed as Māori or non-Māori. The area-based measure used was the NZDep2001 Index of Deprivation [16]. This combines nine variables measured in the 2001 Census which reflect aspects of social and material deprivation; each Census meshblock has been allocated a deprivation score. In the current study, the area-based SES was then determined by geocoding each adolescent's street address and matching it (via meshblock number) to the NZDep01 data-base. For intra-examiner reliability, the intraclass correlation coefficient for DMFS was 0.96; for inter-examiner reliability, it was 0.97. Ethical approval was obtained from the Lower South Ethics Committee.

### Brunei

A cross-sectional epidemiological survey of Year-6 schoolchildren (age 10 to 14) attending the nine government primary schools in Brunei Zone II (Brunei-Muara district) was conducted in 2010. A Malay version of the short-form CPQ was derived through a forward-backward translation process, then piloted and adapted. Ethnicity information was collected from the parent/caregiver. Information on the parent/caregiver's occupation was recorded from the consent form. Household SES was then determined using the Malaysia Standard Classification of Occupations (2008). For intra-examiner reliability, the intraclass correlation coefficient for DMFS was 0.99; for inter-examiner reliability, it was 0.99. Ethical approval was obtained from the Medical and Health Research and Ethics Committee, Ministry of Health, Brunei.

### Brazil

A cross-sectional study was conducted involving 11- to 14-year-old schoolchildren in public and private schools from 13 municipalities in the Midwest Region of the Brazilian Southern State of Santa Catarina in 2009. Non-clinical data were collected through structured interviews, and included sociodemographic characteristics, including sex of the child and one measure of socio-economic status (whether the father was currently working). Ethnicity data were not collected. The reproducibility of clinical diagnosis was tested through duplicate examinations on 10% of the sample by each of the examiners; this showed Kappa values (both intra- and inter-examiner) greater than 0.8, calculated on a tooth-by-tooth basis. The project obtained approval by the Ethics Committee of the *Universidade do Oeste de Santa Catarina*, Brazil.

Data were analysed using the Statistical Package for the Social Sciences (version 18). Missing responses for any item was allocated a score of zero at analysis stage. Children who presented in the 20% with the greatest caries experience (DMFS score) were categorised as the highest caries quintile (this ranged from DMFS = 4+ in Otago and Brunei to DMFS = 8+ in Northland). Following the computation of univariate descriptive statistics, differences among proportions were tested for statistical significance (P < 0.05) using chi-square tests; differences among means were tested for statistical significance (P < 0.05). Construct validity was evaluated by comparing the mean scale scores across the categories of caries experience using Mann-Whitney or Kruskal-Wallis tests (as appropriate). The alpha value was set at P < 0.05. Correlational construct validity was assessed by comparing mean scores and children's global ratings of oral health and well-being using Spearman's correlation coefficient.

## Results

Data on the characteristics of the four samples are presented in Table 1. Sample size ranged from 187 (Northland, New Zealand) to 457 (Brunei), with broadly similar age ranges (with the Brunei and Brazil data including 10- and 11-year-olds). Males comprised approximately half of the participants in each sample. For ethnicity, the New Zealand children were classified as Māori or non-Māori, with the Northland sample having nearly three times more Māori than that from Otago. The Brazil children were all classified, as 'Brazilian' and most of the Brunei children were Malay. Mean DMFS score (Table 1) ranged from 4.9 in Northland to 1.8 in Otago. The DMFT score of the Brazilian sample was similar to the DMFS score for Otago.

**Table 1:** Characteristics of participants by study

	Northland	Brunei	Brazil	Otago
Sample size	185	423	404	272
				
Age range	12-13	11-14	11-14	12-13
				
No of Females (%)	89 (48.2)	217 (51.3)	199 (49.3)	127 (46.7)
				
Mean DMFS (SD)	4.9 (5.2)	2.0 (3.8)	1.8 (2.1)^a^	1.8 (3.2)
				
Type of sample	Convenience	Convenience	Convenience	Convenience
				

^a^Surface-level data were not available for the Brazil sample

### Construct validity

**Figure 1:**
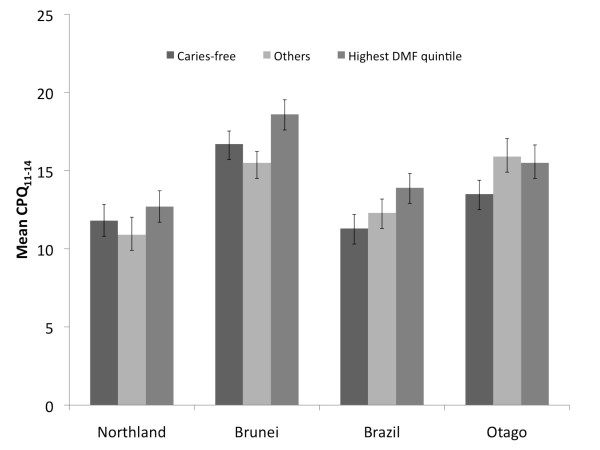
**Mean CPQ_11-14 _and caries experience by sample (caries-free in solid black; others in light grey; highest quintile in dark grey)**.

The CPQ_11-14 _short-form of the questionnaire detected differences by caries experience (Figure 1) in each of the samples, with the mean scores for the highest-caries quintile group greater than those for the caries-free children. The smallest difference was observed in the Northland sample. Overall, the mean CPQ_11-14 _score was highest for the Brunei sample followed by Otago with the lowest in Northland. The mean CPQ_11-14 _and domain scores differed in all of the samples (Table 2) while the relative contribution of the domains ranged from 17 to 39%. The Brunei sample had the highest overall CPQ_11-14 _score and presented with the greatest relative contribution from the social well-being domain. The Northland sample presented with the greatest DMFS score and had the greatest relative contribution to the CPQ from the oral symptoms domain.

**Table 2:** Mean ISF 16-item CPQ_11-14 _scores and their relative contribution (SD)

	**CPQ**_**11-14 **_**(95% CI)**	Range of scores	**CPQ**_**11-14 **_**domain scores**				**Relative contribution to overall scale (%)**^**a**^			
		
			OS	FL	EW	SW	OS	FL	EW	SW
Northland	11.5 (7.3) 10.4 - 12.6	1 - 40	4.5 (2.5)	2.2 (2.3)	2.6 (2.5)	2.4 (2.5)	39	19	22	20
Brunei	16.8 (8.7) 16.0 - 17.6	0 - 43	5.0 (2.6)	3.8 (3.0)	4.3 (3.0)	3.7 (2.7)	30	22	26	22
Brazil	12.4 (9.2) 11.5 - 13.3	0 - 49	4.1 (2.6)	2.8 (2.9)	3.4 (3.5)	2.1 (2.5)	33	23	27	17
Otago	14.6 (8.6) 13.6 - 15.6	0 - 40	4.7 (2.3)	3.4 (3.0)	3.7 (3.1)	2.8 (2.7)	32	23	25	19
										

^a ^OS = Oral Symptoms, FL = Functional Limitations, EW = Emotional Well-being, SW = Social Well-being

All forms of the CPQ_11-14 _showed greater scores in groups with worse self-reported oral health (Table 3). A consistent gradient was observed in the scores across the response categories from 'Not at all' to 'A lot/Very much' with the impact on quality of life for all except for the Brunei sample. A similar gradient was observed for the self-rated oral health responses 'Excellent' to 'Fair/Poor' except for the Northland and Brunei sample. All samples demonstrated positive, statistically significant and similar correlations with the ratings of oral health and overall impact on quality of life, although it was lowest in the Brunei sample.

**Table 3:** Construct validity: performance of CPQ_11-14 _versions against global questions

Global Questions	**CPQ**_**11-14 **_**ISF16(SD)**			
	**Northland**	**Brunei**	**Brazil**	**Otago**

				
Self-rated oral health				
Excellent	9.4 (4.2)^a^	15.6 (7.6)^a^	7.4 (6.0)^b^	9.6 (6.7)^b^
Very good	9.1 (6.8)	16.5 (9.0)	8.3 (5.7)	10.9 (7.0)
Good	11.4 (6.8)	15.6 (8.3)	9.4 (7.2)	14.1 (7.3)
Fair/Poor	16.3 (9.0)	18.7 (9.0)	15.7 (10.0)	19.7 (10.1)
Spearman's rho^c^	0.28	0.11^d^	0.38	0.37
				
Impact on quality of life				
Not at all	8.9 (5.7)^a^	13.89 (8.3)^a^	7.6 (6.1)	10.7 (5.6)^b^
Very little	12.4 (6.6)	16.3 (9.1)	13.8 (8.8)	13.9 (7.1)
Some	14.6 (7.5)	18.5(7.7)	16.2 (10.3)	18.2 (9.3)
A lot/Very much	15.3 (0.9)	17.8 (9.8)	17.4 (10.2)	24.8 (12.3)
Spearman's rho^c^	0.32	0.19	0.39	0.37
				

^a ^p-value < 0.05 Kruskal-Wallis/Mann-Whitney

^b ^p-value < 0.01 Kruskal-Wallis/Mann-Whitney

^c ^correlation significant at 0.01 level

^d ^correlation significant at 0.05 level

## Discussion

Validation of the short-form measures of the CPQ_11-14 _at the population level is important, because clinical studies may give a misleading picture because of the biased nature of their samples [17]. This study of the performance of the short-form version of the CPQ_11-14 _among children from four different communities with differing caries experience has found that the short-form version of the CPQ_11-14 _performs well in terms of validity. However, the observed differences in mean scores across the samples need further exploration in order to fully understand what this phenomenon represents.

Before discussing the findings, it is appropriate to consider the study's weaknesses and strengths. The non-representativeness of all of the samples is a weakness, because it means that the generalisability of the findings is limited. On the other hand, the relative uniformity of findings in convenience samples from a number of different communities within New Zealand and internationally is a strength, in that it suggests that the short-form version has validity in different settings and populations. Among the study's other strengths was that the short-form version was administered to adolescents prior to being clinically examined in all the samples as well as the comprehensiveness of the data collection (with caries data collected at surface level rather than tooth level, for all but one sample) with examinations conducted under acceptable conditions by calibrated dentists in public health settings rather than in other, more *ad hoc *settings.

The construct validity of the short-form version is supported by its ability to detect differences in quality of life, evident in the highest scores being seen in the children with the greatest caries burden. A clear difference did exist, with greater mean CPQ_11-14 _scores in children presenting with the greatest caries experience relative to those who were caries free, and this held irrespective of the community. Concerning dental caries experience, there were distinct CPQ differences (in both the overall and the domain scores) between those who were in the highest quartile for DMFS and the remainder. These findings are not counter-intuitive: other factors being equal, children in the most severe disease quartile are likely (for example) to have experienced more oral pain, had difficulties in chewing, to have worried or been upset about their mouths, or to have missed school due to their cumulative disease experience [1].

Variations among populations were apparent, with Brunei children reporting higher scores (indicating a greater impact on their OHRQoL). Even within the same country (New Zealand), variations existed. These appear not to be related to overall caries experience. Comparing samples, there appears to be no clear association between mean CPQ score and caries experience, as the sample with the greatest caries burden did not have the highest mean CPQ score. The Northland children had more than twice the caries burden of those from Brunei, Brazil and Otago, but this was not reflected in their overall mean CPQ score. However, they did have the greatest relative contribution of the oral symptoms domain to that score.

The earlier reported New Zealand study using the 37-item questionnaire to evaluate the short-form version had a lower mean score than either of the two New Zealand samples in this study. This could be due to the possibility that the children may have responded differently when answering the longer questionnaire. However, an Australian study found no significance differences in scores when the short-form 12-item health survey version was embedded in the longer-form 36-item version as opposed to administering it separately to an equivalent representative sample [18]. The current study shows that there were different overall scores (even with both samples having the short-form self-administered) in the two New Zealand samples, and it is more than likely the difference in scores may reflect differences in the populations of adolescents in the New Zealand regions. This was not reflected in the current Brazil sample, as its mean CPQ score was very similar to the earlier reported Brazil study with the mean scores (12.4 and 12.9 respectively) differing by a small amount [13]. This similarity may reflect heterogeneity in the Brazilian population which does not occur in New Zealand, or it could be an artefact, and if another Brazilian community was sampled, a different mean CPQ score could occur, as is the case in New Zealand.

This variation within and between countries makes cross-cultural comparisons using mean CPQ scores difficult to interpret. This has already been found with the 14-item Oral Health Impact Profile (OHIP) when comparing oral disorders in the United Kingdom and Australia, with dentate Australians reporting a higher number of impacts than dentate United Kingdom adults. These differences may have reflected subtle socio-cultural differences in subjective oral health among these populations [19] and could similarly account for the differences in our samples under study. They also surmised that these subtle differences can tell us quite a lot about the social and psycho-social influences on oral health-related quality of life between populations and among sub-groups within populations. In an earlier study of older people in South Australia, Ontario and North Carolina, smaller differences were observed between countries than between different racial groups within countries [8]. This sort of effect may account for the differences in the Northland and Otago communities, with over two-thirds of the Northland sample (but fewer than the one-fifth of the Otago sample) being Māori.

The case for construct validity is further supported by the assessment of the short-form of the CPQ_11-14 _against the global questions. All of the samples demonstrated positive and significant correlations with both global questions, as observed in the recent studies reporting on short-form versions (13,15) and all samples had a higher score in those with poorer oral health. Overall, mean scale scores were greater for those reporting 'Fair/Poor' self-rated oral health than for those reporting 'A lot/Very much' impact on their quality of life. In developing the short-form versions, Jokovic and co-workers predicted that, in evaluating construct validity, the correlation coefficient would be higher for the rating of well-being than for the rating of oral health, because the former is a measure of health-related quality of life and the latter a measure of health (11). This had been shown in the longer questionnaire and was borne out in the Toronto clinical convenience sample data (13) and the earlier New Zealand population sample (14), although it was not reported with the two item-impact short-form versions administered in Brazil (15). This meant that the smaller number of items in the short-form version might compromise its construct validity. In the current study, higher correlations were reported between the CPQ and well-being than for self-rated oral health in nearly all of the samples (Otago had the same score). This reinforces the fact that the items in the short-form also address issues and concerns that go beyond oral health and are of sufficient magnitude to have some effect on life as a whole [17]. This confirms that the smaller number of items in the short-form version does not compromise its construct validity.

The current study confirms that the 16-item short-form impact version of the CPQ_11-14 _performs well across diverse cultures and levels of caries experience. Differences in mean CPQ scores between the communities may reflect subtle socio-cultural differences in subjective oral health between these populations but elucidating these requires further exploration of the face and content validity of the measure in different populations. Further population-based research is required in order to further explore the cross-cultural utility of the CPQ_11-14 _and the underlying importance of the measure.

## Competing interests

The authors declare that they have no competing interests.

## Authors' contributions

LFP carried out the New Zealand data collection at two sites, analysed the data from all populations and drafted the manuscript. WMT participated in study design, and helped draft the manuscript. ARM collected the Brunei data and analysed and JT collected the Brazilian data with analysis. All authors read and approved the final manuscript.
